# Thoughts from the forest floor: a review of cognition in the slime mould *Physarum polycephalum*

**DOI:** 10.1007/s10071-023-01782-1

**Published:** 2023-05-11

**Authors:** Chris R. Reid

**Affiliations:** School of Natural Sciences, Macquarie University, North Ryde, NSW 2109 Australia

**Keywords:** Basal cognition, Minimal cognition, Extended cognition, Embodied cognition, Non-neural, Collective behaviour

## Abstract

Sensing, communication, navigation, decision-making, memory and learning are key components in a standard cognitive tool-kit that enhance an animal’s ability to successfully survive and reproduce. However, these tools are not only useful for, or accessible to, animals—they evolved long ago in simpler organisms using mechanisms which may be either unique or widely conserved across diverse taxa. In this article, I review the recent research that demonstrates these key cognitive abilities in the plasmodial slime mould *Physarum polycephalum*, which has emerged as a model for non-animal cognition. I discuss the benefits and limitations of comparisons drawn between neural and non-neural systems, and the implications of common mechanisms across wide taxonomic divisions. I conclude by discussing future avenues of research that will draw the most benefit from a closer integration of *Physarum* and animal cognition research.

## Introduction

Animals can acquire information from their environment through their senses, remember it, use it to solve problems and learn, anticipate and communicate, all of which serves to increase their fitness in a world that is complex, dynamic and dangerous. Animals (at least those traditionally associated with being ‘cognitive’) perform these feats using the highly sophisticated information-processing hardware that we call a brain. These facts are well established, and few schoolchildren (and still fewer cognitive scientists) would challenge them. Similarly, few would argue that organisms without brains—forming the vast majority of current and past life on this planet—live in some parallel world that is homogeneous, static and safe. Since all organisms face similar environmental challenges, and thus stand equally to benefit from the adaptive advantages of detecting, storing, and learning from environmental information, it is strange that the word most often used to describe this function—cognition—has traditionally been strictly reserved for the historically recent, neurally equipped minority.

The belief that cognition requires a nervous system remains pervasive, but faces continued and growing challenge (Allen 2017; Lyon et al. 2021; Solé et al. 2019). This groundswell has led to a formal framework called *basal cognition* for reframing the definition of cognition, based on the premise that complex cognitive function had to evolve from earlier, simpler systems, rather than beginning with assumptions of (typically human) uniqueness and working ‘down’ the evolutionary tree (Lyon et al. 2021). This method, the authors argue, would simply bring the cognitive sciences into alignment with all other life sciences in taking a bottom-up approach that begins with looking at the smallest, simplest, earliest-evolved organisms to display phenomena of interest, identifying the principles of function, and from there scaling up to more complex organisms, comparing similarities and differences.

One of the difficulties of the bottom-up approach is that there is an awful lot of bottom to choose from, with small, simple organisms occupying every conceivable niche that could support life. While the majority may face similar fundamental environmental challenges, peculiarities of their evolutionary past, life history, and physiology make it likely that their evolved mechanisms for responding to their world are as diverse as the organisms themselves. Naturally, research has been limited to those particular ‘basal’ organisms that are easy to culture in the lab, and can be rigorously tested with behavioural experiments to which they unambiguously and reliably respond. This has included bacteria (Lyon 2015; Shapiro 2007), plants (reviewed in Segundo‐Ortin and Calvo 2022), protists (e.g. *Paramecium* (Armus et al. 2006; Gelber 1957; Gershman et al. 2021), *Amoeba* (De la Fuente et al. 2019) and *Stentor* (Bennett and Francis 1972; Rajan et al. 2022)), and simple neural animals such as planaria (Prados et al. 2013, 2020) and Cnidaria (Jennings 1905; Logan 1975). One non-neural model system in particular has recently shown consistent and unambiguous success in demonstrating a range of cognitive functions: the slime mould *Physarum polycephalum*.

The purpose of this review is to introduce *Physarum polycephalum* to the general readership of animal cognition research, highlighting recent and historical work that demonstrates basal cognitive capacities in this fascinating organism. One key goal is to elucidate the hypothetical mechanisms of various cognitive functions in this single-celled protist, to guide future comparative approaches that span a greater breadth of the phylogenetic tree. Hopefully, this will lead to a greater understanding of the evolution of cognition; it will at the very least reveal some of the great diversity of biological approaches to making sense of the world.

## Physarum biology

**Fig. 1:**
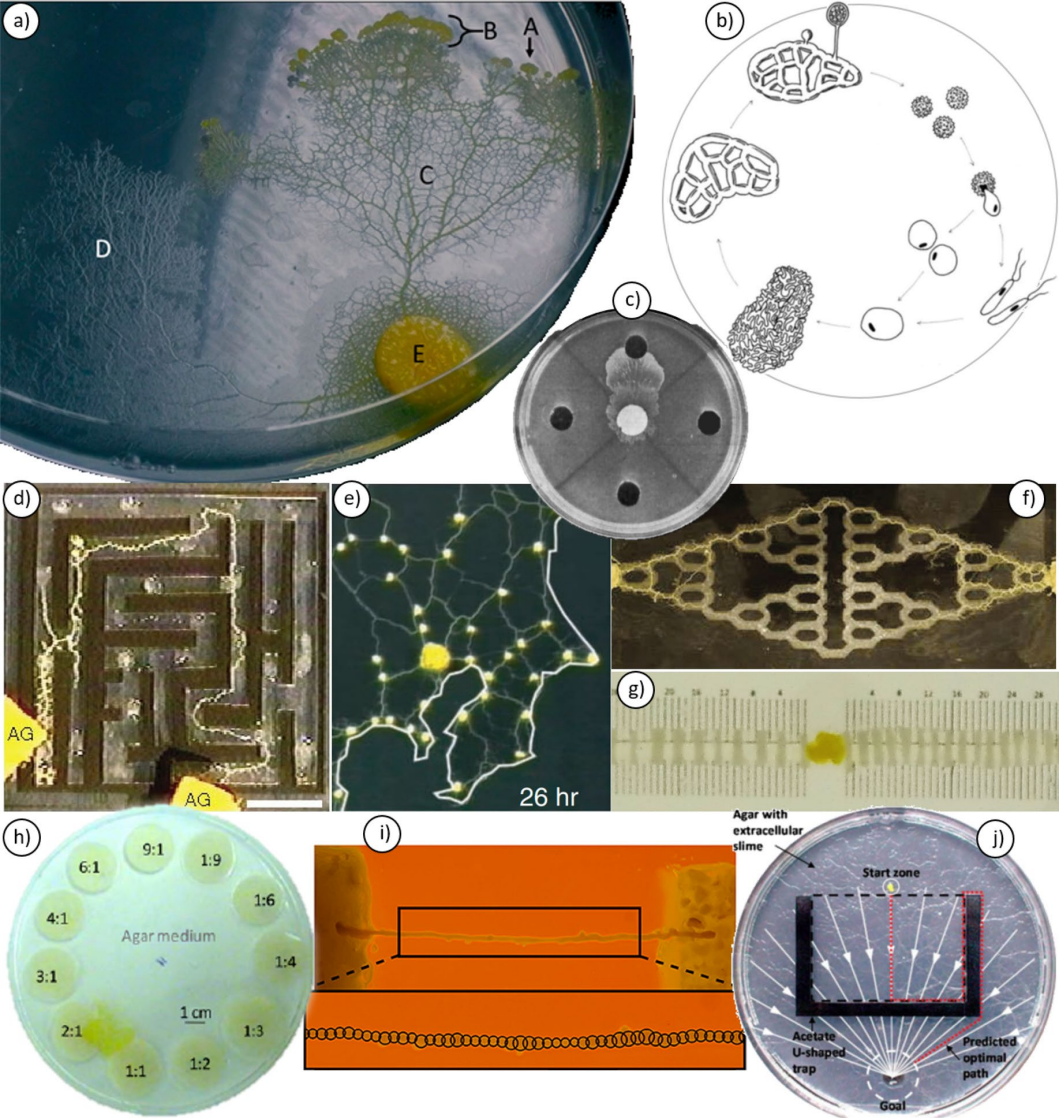
**a** Features of the plasmodial stage of Physarum, including (A) extending pseudopod, (B) search front, (C) tubule network, and (D) extracellular slime remaining from areas previously explored. Food disk containing original inoculation depicted at (E). E is approximately 0.5 cm in diameter for scale (Reid et al. 2012). **b** Life cycle of Physarum clockwise from haploid spores (top right) which hatch to form free-living amoeboid or flagellated cells. These can fuse to form a diploid microplasmodium, which grows into a mature plasmodium that produces sporangia when starved or stressed (adapted from (Reid and Latty 2016). **c** Quadrant test for chemotaxis towards glucose (top well, central disk is approximately 1 cm in diameter for scale, Knowles and Carlile 1978). **d** Plasmodium finding the shortest path through a labyrinth maze (scale bar 1 cm, Nakagaki et al. 2000). **e** Plasmodium connecting oat food sources on a map of the Tokyo railway network (horizontal width of panel 17 cm for scale, Tero et al. 2010). **f** Plasmodium solving the Towers of Hanoi maze (experiments in Reid and Beekman 2013, image from Ma et al. 2013, width of panel approximately 25 cm for scale). **g** Plasmodium (centre) about to solve the two-armed bandit problem, first exploring both arms of agar sites presented to it (right and left), encountering different food distributions along the way. The point at which the plasmodium stops exploring one arm to focus on the other informs us of the organism’s decision-making strategy (distance between neighbouring vertical lines in image 1 mm, Reid et al. 2016). **h** Plasmodium occupying a combination of food options to reach its ideal protein:carbohydrate ratio intake, thereby solving a complex foraging trade-off (Dussutour et al. 2010). **i** A single tubule of plasmodium stretched between two different food sources (separated by 2 cm for scale) to examine the oscillation patterns of 50 equidistant points along the tubule’s length (lower rectangle), in search of decision-making mechanisms (Ray et al. 2019). **j** Testing for use of extracellular slime as an externalised spatial memory: a plasmodium (yellow blob in ‘Start zone’) must navigate towards a diffusing source of glucose attractant, while escaping the U-shaped trap that blocks its direct progress. In this case, the agar surface is coated in extracellular slime, preventing this individual from ‘remembering’ where it has explored, as its own trail is lost in the background (petri dish 8.6 cm diameter for scale, Reid et al. 2012). All images reproduced with permission and/or under licence

*Physarum polycephalum* (hereafter Physarum) is a unicellular eukaryote currently placed in the kingdom Protista. The vegetative stage of Physarum’s life cycle (Fig. 1a, b) is called a plasmodium, which is a single cell containing millions of nuclei flowing freely inside a motile network of protoplasm. The multinucleate nature of Physarum means that individual fragments removed from a plasmodium will self-organise into independent clones of the parent cell, a process that takes only minutes (Kobayashi et al. 2006). Under ideal conditions, a plasmodium can grow as large as 900 cm^2^ in size. It moves in an amoeboid fashion, by extending pseudopods, at speeds of up to 5 cm/h (Kessler 1982) to explore its environment and engulf its prey of bacteria, yeasts and micro-particles. The exploratory ‘search front’ of the organism is a dense fan shape (Rusch 1980). Behind the search front, however, cell morphology is organised into a network of intersecting tubules (Fig. 1a). Within the tubules, protoplasm circulates back and forth rhythmically, with a period of around 1–5 min depending on internal state (Wohlfarth-Bottermann 1979). This constant internal flow transports biomass, nutrients, chemical signals and information (Collins and Haskins 1972), and is coordinated by oscillatory and biochemical mechanisms, which have been the focus of much of the Physarum literature.

## Physarum’s cognitive capabilities

### Sensing

As a free-living organism, the plasmodium is equipped with a range of surface receptors and internal mechanisms for sensing and responding to the world around it. One of the key response mechanisms, underpinning the majority of cognitive, locomotive and homeostatic functions, is oscillation (Durham and Ridgway 1976). The plasmodium can be considered a connected mass of multitudes of tiny oscillating units, capable of expanding and contracting in response to local sensory stimuli via actin-myosin interactions—the same contractile mechanism in human muscle tissue. When cell surface receptors detect attractants such as food and moisture, oscillation frequency increases in the local area, which decreases cell surface tension, making the plasmodium more fluid (Ueda et al. 1980). This causes protoplasm to flow towards the stimulus area, and directs the movement of the entire cell. Repellents such as light and certain salts induce the opposite response, increasing the local stiffness of the cell and restricting further flow into the area.

Much of the cognitive behaviour observed in Physarum is fundamentally a consequence of communication between the myriad contractile units. While each unit senses and responds to the environment around it, physical coupling between adjacent oscillators entrains them to each other’s frequencies (Nakagaki et al. 1999). This means they can respond to and influence the behaviour of their neighbours, and transfer information about the quality of local environments to distant parts of the cell. For instance, in the construction of efficient tubule networks between multiple food sources (discussed in further detail below), attractants such as food and moisture are locally sensed, resulting in increased oscillation frequency in nearby contractile units. Physical coupling results in waves of contraction that propagate outwards from the stimulus area and communicate information between proximal and distal parts of the organism. Network tubules that lie perpendicular to the direction of contractile propagation receive a lower flux of protoplasm and thus begin to decay, while parallel tubes receive more protoplasm and are reinforced and thickened. The tubules able to accommodate the highest flux are those that link the network via the shortest path; network length is thereby optimised via a positive feedback loop (Nakagaki et al. 2000; Tero et al. 2006). This simple method of local communication illustrates a form of distributed collective behaviour that leads to the emergence of sophisticated properties at the organism level (Reid and Latty 2016).

More recent work has focussed on the evidence for a biochemical signal propagated through the organism by the peristaltic waves themselves (Alim et al. 2017). A signalling molecule (later called a ‘softening agent’ (Kramar and Alim 2021)) was found to travel along with the internal flow, increasing contraction amplitude as it travels, and thereby facilitating its own transport via positive feedback. The signalling molecule is unknown but is likely to be either ATP or calcium ions, which are known to be vital for actomyosin interactions. It is important to note that a self-reinforcing signalling molecule could only work for attractant responses in Physarum, which induce fluidity, and not for responses to repellents, which increase stiffness. There is no reason that biochemical and coupled-oscillator models should be mutually exclusive, however, and most likely the two mechanisms are both exploited in Physarum’s toolkit for sensing and responding to the world.

Due to Physarum’s early popularity as a model for cell motility, there is a host of previous studies into positive and negative taxes in plasmodia responding to gradients of carbohydrates, proteins, amino acids, free nucleotides, volatile organic chemicals, salts, pH, light, humidity and temperature (Fig. 1c; (de Lacy Costello and Adamatzky 2014; Chet et al. 1977; Kincaid and Mansour 1978; Knowles and Carlile 1978; Ueda et al. 1980). There is also evidence that Physarum can sense and respond to the direction of gravity (geotaxis (Wolke et al. 1987)), magnetic fields (magnetotaxis (Shirakawa et al. 2012)) and even use mechanosensation to detect heavy masses at long-range (Murugan et al. 2021). Thus, a broad base of literature supports Physarum’s ability to sense, and adaptively respond to, diverse information acquired from its environment.

### Communication

Communication between organisms is generally defined as requiring both a signaller and a receiver, where a signal is “any act or structure that alters the behaviour of other organisms, which evolved because of that effect, and which is effective because the receiver’s response has also evolved” ((Maynard Smith and Harper 2003), p. 3). A far less stringent definition of communication might also include the use of ‘cues’, which are essentially any useful bits of information in the environment, even those originating from other organisms, but which need not have evolved specifically to alter the behaviour of others (Maynard Smith and Harper 2003). There are relatively few studies of communication between slime moulds, either directly or indirectly (let alone making the distinction between ‘clonemates’ originating from the same culture, or distinct individuals), and these few studies have all been framed in a cooperation/competition context.

In the absence of evidence for signalling, studies have focussed on Physarum’s response to cues left behind in the extracellular slime (ECS) trails of clonemates, conspecifics (originating from different cultures or strains), or other species of plasmodial slime moulds. The results indicate a rich repertoire of responses to multiple sources of information about the presence and state of other individuals. Physarum will generally avoid the ECS of clonemates, which thereby acts as a source of externalised spatial memory (see “Memory” section below) and prevents it from wasting energy foraging in overexploited areas (Reid et al. 2012). This avoidance response, however, is context-specific, and can be overridden in certain scenarios, for instance when food source cues are detected in high concentration (Reid et al. 2013). Similarly, while Physarum plasmodia will actively avoid environments previously explored by starved or stressed clonemates, they are attracted to environments previously explored by well-fed clonemates (Briard et al. 2020). Hence, information about the potential foraging value of an environment can pass between individuals. *Physarum rigidum* can recognise conspecific plasmodia by cues within the extracellular slime and chooses to fuse with individuals collected from nearby geographic regions (hence more likely to be allogeneic, and characterised as ‘self’), while avoiding individuals originating from more distant locations (Masui et al. 2018). With the option to fuse its body with that of other individuals, and the complex fitness consequences of doing so, Physarum has clearly evolved a range of mechanisms for sensing and responding to conspecifics.

Physarum can also recognise the presence of ECS originating from other species of slime mould and exhibits a similar contextual response to this information. When encountering the ECS of *Didymium bahiense*, Physarum will prefer to explore elsewhere, unless the only other available choice is over terrain marked with clonemate ECS (Reid et al. 2013). This can be interpreted as the slime mould making a choice between an area that is likely to have been stripped of preferred resources (by either itself or conspecifics), or another that may contain more resources, due to slight differences in heterospecifics’ food preferences. This ‘eavesdropping’ behaviour is similar to that observed in animals such as bumblebees, which not only recognise and reject exploited flowers marked by their own pheromone, but also those marked by other bumblebee species (Goulson et al. 1998).

Only a few studies have placed multiple plasmodia together in the same space at the same time. In one recent study two clonemates placed in the same petri dish containing a single food source had a shorter latency to begin foraging in the presence of clonemates (Stirrup and Lusseau 2019). Foraging began soonest when the focal plasmodium was starved and the clonemate was satiated. While these results were interpreted in a competitive context, they align with the results of a subsequent study (Briard et al. 2020), which found an attraction towards environments previously explored by well-fed clonemates. These results suggest that rather than exhibiting purely competitive responses to conspecifics, Physarum has the potential to exhibit facilitative responses that hint at an as-yet hidden world of social interactions with clonemates.

### Orientation and navigation

While taxis responses (such as chemotaxis) and navigation both involve an organism moving from one place to another, the taxis examples above require only the ability to orient in space. For a macroscopic organism such as Physarum, the ability to sense an environmental gradient and move along it is a relatively simple feat. Many organisms are faced with the cognitive challenge of moving along a route between multiple points, and Physarum can achieve this in two ways. The first is conventional navigation, where an entire plasmodium must migrate through space to reach a distant point. I would argue, however, that the slime mould’s method of exploratory network construction, followed by optimisation of the path between points of interest, is analogous to path-planning strategies utilised by autonomous systems, and hence can be classified as navigation.

Mazes have a long history of use in animal cognition experiments, and these are framed almost exclusively in a learning context (reviewed in Kabadayi et al. 2018). Physarum too has been tested in classic labyrinth mazes requiring the whole organism to move or extend through the maze towards a goal. However, these have never been framed in terms of learning (individuals are never challenged with solving the same maze more than once) and are always investigations of Physarum’s navigational ability. For instance, Adamatzky (2012) placed a food source at the centre of a circular labyrinth maze with an agar floor, challenging the plasmodium to navigate towards the goal. In this case, the walls of the maze extended below the level of the agar, creating a distinct channel of agar along which chemoattractants can diffuse from the centre to the plasmodium inoculation site. It is therefore no surprise that the slime mould simply follows this gradient, and in doing so follows the shortest path through the maze.

Other maze-navigation studies have relied on a subtly different technique whereby the agar substrate is undisturbed by the maze barriers, which are constructed of thin acetate sheets laid on top of the agar surface. As Physarum avoids the hydrophobic surface of the acetate, these barriers prevent Physarum movement, while allowing food cues to diffuse in a continuous radial gradient from the food goal. Thus, the organism must make local navigational choices based on a more-or-less global directional preference, in some cases having to override local chemotactic preference to reach the goal. These problems can be more difficult for Physarum to solve, yet they remain tests of navigation by chemoattraction (Smith-Ferguson et al. 2017, see “Memory” section below discussing the results of this study).

The more commonly researched example of navigation is experimentally framed around Physarum’s excellent ability to construct efficient networks between multiple points of interest. The plasmodium spreads itself out in search of food (exploration), often finding multiple food sources simultaneously some distance apart. This stage is analogous to the slime mould building a map of its surroundings, with the plasmodial network itself forming the many potential routes between food sources A and B. Having found multiple food sources, the plasmodium now seeks to engulf them with biomass, while also staying connected as a single entity (exploitation). This fundamental trade-off has resulted in strong selection for shortest-path-finding strategies in Physarum, which has been a major focus of slime mould behavioural research within the last two decades.

The landmark study which single-handedly spurred this flurry of research activity is a brief communication in *Nature* by Nakagaki and colleagues (2000), in which Physarum plasmodia were spread through a labyrinth maze connecting two food sources, forming a single cell in the shape of the maze (Fig. 1d). Over time, the plasmodia retracted biomass from the dead ends and longer paths through the maze, until eventually a single tubule remained, tracing out the single solution. As stated by the authors, “This remarkable process of cellular computation implies that cellular materials can show a primitive intelligence.” (Nakagaki et al. 2000). These humble words sparked a revolution, and a host of studies followed, exploring the capabilities of Physarum in network optimisation (Nakagaki et al. 2001, 2004; Reid and Beekman 2013), and building mathematical models based on empirical insights (Tero et al. 2006, 2007).

These studies have been of particular interest to network engineers seeking new methods for designing optimisation algorithms. Human-designed networks, such as those for telecommunication or transport, tend to place a high priority on shortest paths, as these provide the quickest travel time through the network, and the lowest construction cost. These networks are, however, the most at risk of catastrophic failure after even the slightest disruption, so natural selection has favoured biological networks that find a trade-off between path efficiency and the robustness of additional redundant links (Middleton and Latty 2016). A landmark study produced by Nakagaki’s lab (Tero et al. 2010) sought to extract network design rules from Physarum that specifically encoded an optimal trade-off between shortest-path efficiency and robustness. The researchers allowed Physarum to explore an agar plate in the shape of the Tokyo district, with food sources placed in the locations of railway stations (Fig. 1e). As the slime mould pruned its network to connect these ‘stations’ (over the course of approximately 26 h), the resulting networks had comparable efficiency, fault tolerance and cost to the existing Tokyo Railway infrastructure. Moreover, the authors were able to use the behavioural observations of Physarum network construction to build mathematical models for network growth, with tuneable parameters for adjusting features such as fault tolerance and transport efficiency. These could be useful for engineers designing future transport networks, or for guiding the development of self-organised networks such as remote sensor arrays, wireless mesh networks, or the Internet-of-Things (Tero et al. 2010; see also Gao et al. 2019 for an extensive review of Physarum-inspired models and computations, and their impacts).

While these feats of optimisation are impressive, they present to the organism an ideal situation of a static problem in which the environment never changes. Biological systems have been selected to survive in dynamic environments, and so must have evolved mechanisms for solving this difficult class of problems. In my own previous work (Reid and Beekman 2013), we used a well-known shortest-path problem—The Towers of Hanoi maze—to test Physarum’s dynamic problem solving in a maze-navigation scenario (Fig. 1f). When allowed to build a network through the Towers of Hanoi maze, Physarum occasionally constructed minimal length paths, but often built longer networks, in contrast to the results of Nakagaki’s first labyrinth maze. This may be because the Towers of Hanoi maze was much more difficult, having 32,678 unique paths through the maze, compared to the Nakagaki maze’s 4 possible solutions. However, when the Towers of Hanoi maze shape was changed following Physarum solving it, the plasmodium often retracted fully from the maze and then flowed through it anew, building new connections and pruning its network. Upon solving the new maze, Physarum was much more efficient, always building networks with minimal-length paths. In response to these dynamic changes, Physarum was able to utilise some sections of its original network, and construct large sections of the network from scratch, perhaps being guided by its aversion to previously laid ECS to find the optimal solution space. We compared Physarum’s solution strategy to another distributed biological system that has been tested with the same maze—ant colonies (Reid et al. 2011)—finding that the stronger reliance on positive feedback ensures that ants are more likely to converge quickly on the optimal solution to a maze, but at the cost of being less adaptable to dynamic changes than Physarum, which likely relies less on positive feedback (Reid and Beekman 2013).

### Decision-making

Decision-making is defined here following Reid et al. (2015) as “the action by an entity of selecting an option from a set of alternatives, based on characteristics of the alternatives that the entity can perceive.” The utility of this definition is that it relies only on the observable actions of the organism in question, and makes no assumption of underlying mechanisms. As Physarum can clearly perceive a vast array of information about its environment, it is no surprise that plasmodia consistently choose the better of two presented options when they differ in only a single attribute, such as caloric concentration (Latty and Beekman 2010), temperature (Durham and Ridgway 1976) or light levels (Latty and Beekman 2011b). Indeed, most of these decisions can be made using simple taxis responses. An exception is a study of length discrimination (Mori and Koaze 2013). When presented with the option of connecting two food sources via either a short or long route in a circular arena, Physarum predictably chose the shorter route. This choice was consistent despite changing the diameter of the arena, indicating that Physarum bases its decision on the ratio of the two lengths, rather than any absolute difference. This pattern of cognition of difference in stimulus magnitude (constrained by Weber’s law (Fechner 1948)) is consistently observed in decision-making systems from humans (Deco et al. 2007) to other mammals (Yoshioka 1929), birds (Dixit et al. 2022) and insects (Perna et al. 2012).

Similarly, when examining the decision to remain within a food patch or explore elsewhere, both Physarum and another plasmodial slime mould (*Didymium bahiense*) were found to use incremental patch-departure heuristics, just as insects and humans do (Latty and Beekman 2015). Plasmodia were inoculated inside a grid of circular food discs of either high or low quality, and the time taken to leave the patch was recorded as a function of the number and quality of food sites sampled. Importantly, chemosensory cues of patch quality were precluded by placing each of the food sources on non-permeable plastic circles. Furthermore, the experimental plates were designated as either ‘safe’ (darkened) or ‘risky’ (well lit) environments. Physarum tended to stay longer within a patch if they had recently experienced high-quality food, and within darkened, ‘safe’ patches. *D. bahiense* tended to remain within a patch if it had recently encountered food of any quality and did not alter its strategy in dark or lit environments. Studies such as this highlight the utility of cognitive paradigms applied across (and within) broad taxa, as well as outlining a rich future avenue of research into why and how these diverse strategies exist, even between two species of plasmodial slime mould.

In the interests of understanding cognition, it is necessary to explore more difficult decision-making scenarios, such as when multiple attributes per option can be evaluated independently, and when several of these attributes may conflict with each other. These so-called ‘multi-attribute compensatory problems’ are the gold standard, because they require the organism to compare options based on their relative differences, integrating information along multiple axes of ‘quality’, rather than simply whether one attribute exceeds a desired threshold (see Reid et al. (2015) for a detailed discussion of decision-making in Physarum and other non-neural organisms).

Physarum is capable of making trade-offs between exploitation of food and exposure to danger. For instance, when choosing between high-quality food (positive stimulus) that is illuminated with strong light (negative stimulus), and an alternative option of low-quality food in the dark, Physarum will choose the safer, low-reward option. However, if the risky option is at least five-fold higher in food concentration than the safe option, Physarum will gamble on the illuminated food source (Latty and Beekman 2010). When forced to build a network connecting two food sources that passes through an intensely lit region, Physarum will make the optimum trade-off of path efficiency and exposure to light (Nakagaki et al. 2007). The single tubule connecting the food sources travels along a deflected path that occupies more space within the dark region when the lit region has a higher photo-intensity—a geometric feature similar to the path of light travelling through two materials with different refractive indices (Nakagaki et al. 2007). Importantly, the tubule path did not differ between totally dark and totally lit controls, indicating the amount of deflection is determined entirely by Physarum’s computation of the ratio of light intensity between the two regions.

For reasons of simplicity, most studies of decision-making focus on binary decisions (choosing between only two available options), where the decision to select one option is indistinguishable from the decision to reject the other. Few studies examine ternary or higher-order decision-making. In a notable exception, Physarum plasmodia were given a choice between 3 food sources that were all identical in value (Marshall et al. 2022). When the sum of the quality of all the food sources was low (low ‘magnitude’), plasmodia took significantly longer to make a decision than when the options had high magnitude; an identical result was recorded by Dussutour and colleagues (2019) in a binary-choice scenario. This is evidence for the widespread phenomenon of magnitude-sensitivity (also called value-sensitivity), where decision-makers show faster, less accurate responses when the value of all available options is high (Pirrone et al. 2022). Presented with a simultaneous choice of 11 different diet options, each of which differed in their ratio of protein (P) to carbohydrate (C) although none comprised the P:C ratio known to be Physarum’s ideal (Simpson and Raubenheimer 1993), the plasmodia spread their biomass over multiple options to select a combination that together was closest to their ideal intake target (Dussutour et al. (2010), Fig. 1 h). This is analogous to solving the ‘Knapsack problem’ of combinatorial optimisation, a problem relevant to many real-world processes where, given a set of items of known weight and value, an operator must choose the combination of items that maximises the value of the collection, but remains below a weight limit (Martello and Toth 1990).

While most animal cognition studies focus on adaptive decision-making, where the outcome of the decision-making process is a beneficial response that increases the organism’s fitness, it can be useful to examine maladaptive cases, such as irrational decision-making. Previous explanations of irrationality have centred around neurological mechanistic explanations, such as dopamine-reward systems (Anselme and Güntürkün 2019; Cocker et al. 2012), or specific brain regions such as the ventromedial prefrontal cortex (Koenigs et al. 2007). However, evidence from Physarum suggests that even non-neural organisms can be irrational. When Physarum plasmodia choose between two options that vary equally in two competing attributes—food concentration and light intensity—they show no preference for one option over the other. However, when a third, inferior option is introduced, plasmodia change their preference (Latty and Beekman 2011a). This is irrational because the decoy option should not affect the organism’s choice. In a related study, the same researchers showed that Physarum plasmodia are subject to speed/accuracy trade-offs when faced with difficult decisions (Latty and Beekman 2011b), another phenomenon that has been traditionally explained using a neuroscientific lens (Bogacz et al. 2010; Chittka et al. 2009).

It is clear (though often overlooked) that decision-making in non-neural and neural organisms is based on at least some shared underlying principles. We cannot hope to understand shared mechanisms, however, until we learn more about how decision-making works outside of a brain. To this end, some recent research has focussed on elucidating the mechanisms of decision-making in Physarum. A classic model for examining decision-making in animals is the multi-armed bandit problem, which explores how animals respond to the exploration–exploitation trade-off: should I exploit familiar but potentially sub-optimal options, or risk further exploration to potentially obtain superior ones? The bandit problem is inspired by casino slot machines (one-armed bandits), where gamblers decide which machine to play to maximise net payoff (Gittins 1979). The paradigm has been applied empirically to humans, birds, fish and social insects (Krebs et al. 1978; Keasar et al. 2002; Shettleworth and Plowright 1989; Thomas et al. 1985; Toyokawa et al. 2014) and also to Physarum (Reid et al. 2016).

In the two-armed bandit test, plasmodia were placed between two environments that differed in food site availability and profitability along their length, thereby constituting the ‘arms’ of a two-armed bandit (Fig. 1 g). Plasmodia could extend pseudopodia into each arm to explore them, and then cease exploring one option to favour exploiting the other, once a decision had been made. By providing a range of different testing scenarios, the study demonstrated that Physarum compares the relative qualities of available options, integrates over sequential samplings to perform well in unpredictable environments, and combines information on both reward frequency and magnitude to make correct adaptive decisions (Reid et al. 2016). Increasing the level of difficulty, the researchers proposed 10 different heuristic rules of varying complexity that Physarum potentially could use to accurately exploit information to maximise food intake. These ranged from extremely simple rules (autocorrelation: move in the same direction as the previous timestep) to the only provably optimal method for solving the bandit problem, the Gittins index (select the arm with the highest index, which takes account of future expected rewards from both exploration and exploitation of an arm, based on a Beta prior over its expected Bernoulli reward probability, and a discount parameter applied to future rewards). Comparing the performance of each model to the experimental data via Bayesian model selection, Physarum proved to operate at the mid-level of complexity, where the probability of exploring each arm is proportional to the number of rewards previously encountered on that arm (Reid et al. 2016). This heuristic is computationally far simpler than the provably optimal strategy, yet it performs nearly as well, and can be employed on a fully decentralised basis by reinforcing exploitation of locally sensed areas of profitability in the environment. It is also mathematically and conceptually similar to the matching law (Poling et al. 2011), where relative rates of responding to a stimulus match relative rates of reinforcement for the stimulus—a pattern widely observed in vertebrates from pigeons (Herrnstein 1961) to rats (Sanchis-Segura et al. 2005), coyotes (Gilbert-Norton et al. 2009) and humans (Alferink et al. 2009), when certain reinforcement schedules are applied.

Studies such as those above have gone some way to describing the *how* of Physarum decision-making, but what do we know of the *where*? When examining the physical mechanisms of decision-making in the brain, researchers can determine the location and pattern of electrical signals within and between different brain regions. Ray and colleagues (2019) devised an analogous method to examine the sites of decision-making in Physarum. Using a single tubule of plasmodium stretched between two food sources, the authors measured the amplitude and frequency of contractions at 50 equally spaced locations along the length of the tubule, as the organism decided which food source to exploit (Fig. 1i). Using the information-theoretic measure of Transfer Entropy (Schreiber 2000), the researchers could then determine how different regions of the slime mould responded to the information, and how this information was transferred to other regions. When both food-source options were identical, contractile regions nearest each of the options act as information sources, while those at the tubule midpoint act as information destinations. When food options differed in quality, the regions near the rejected food source transfer information towards the regions near the chosen food source. This appears counter-intuitive to other models of information flow within Physarum, but the authors point out that their results do not indicate whether information transfer occurs via increasing or decreasing contraction properties, and their method does not allow for establishing a direct causal relationship between contraction properties and decision-making. Despite this caveat, the amount of information transferred between tubule regions was fourfold greater when there was a fivefold difference in food source quality, which provides some evidence for a causal link between information transfer and decision-making.

### Memory

In contrast to the many definitions of decision-making, memory has a single generally accepted definition: the means by which information is stored and retrieved (Kilian and Muller 2002; Sweatt 2009). Thus, the phenomenon is widespread among both biotic and abiotic systems, from the brain allowing you to read this article, to the computer I am using to write it. Even so, the majority of cognition research couches memory in a specifically neural context, perhaps in part due to memory being a key prerequisite for that perennial favourite of cognition researchers: learning (discussed below). Thus, it is unnecessarily surprising that Physarum has been demonstrated to exhibit multiple forms of memory.

Tubules within the greater plasmodial network that lie close to a food source were recently shown to be thickened and reinforced, by local release of a softening agent that facilitates its own transport through the network (Kramar and Alim 2021). Local growth occurs at the expense of tubes more distant to the food source, or which flow in directions that do not align with the attractant’s location. This suggests that alteration of tubule properties can act as a form of physical memory encoding the location of a food source, and that this encoded memory can be ‘read out’ upon discovery of a new nutrient stimulus encountered in the same direction. In this context it could be argued that tube thickening equates to ‘memorising’ the attractant’s location, and retraction of tubes that lead elsewhere equates to ‘forgetting’.

Physarum’s ability to sense its own trail of extracellular slime allows it to essentially build up a map of its explored environment. Rather than storing this spatial information inside itself, the information is encoded in the external environment, and retrieved whenever it encounters the trail (Reid et al. 2012). While showing that this memory system is not necessary when navigating in simple environments, the authors demonstrate that when navigating complex environments, such as escaping a U-shaped trap to obtain a food source, Physarum’s ability to utilise its externalised spatial memory dramatically enhances its navigational efficiency (Fig. 1j). Sims and Kiverstein (2022) argue that this constitutes an example of extended cognition, where at least some of the heavy lifting of cognitive processing is performed by entities located within the local environment and external to an agent’s body (Cheng 2018; Clark and Chalmers 1998; Gillett et al. 2022). Furthermore, they suggest that Physarum’s use of ECS (extracellular slime) corresponds to cognitive niche construction, “the process of actively building structures in the local environment that aid learning and problem-solving.” (Sims and Kiverstein (2022), citing (Clark 2008; Wheeler and Clark 2008)).

Physarum’s use of ECS in more complex mazes than a U-shaped trap has been explored in a follow-up study by Smith-Ferguson and colleagues (2017). Small plasmodia were challenged to navigate through (1) ‘open’ mazes with obstacles to avoid, but not arranged in thin bounded channels as in a labyrinth; (2) ‘simple’ bounded labyrinth mazes with only three paths and 2 decision points; and (3) ‘complex’ bounded labyrinths with 3 decision points and several long, dead-end pathways. The experiments were then repeated in mazes with agar pre-coated in ECS to disable the focal plasmodium’s ability to utilise memory. External memory was found to enhance navigational efficiency in the open and simple bounded mazes but not in complex mazes. In the complex mazes, plasmodia that happened to make a choice leading to a dead-end were prevented from quickly retracing their steps to get back on the correct track. Thus, at least in these artificial scenarios invented by experimenters, the simple heuristic of avoiding areas previously explored can be a handicap rather than an advantage.

Physarum can also build up a memory of periodic events and anticipate their predicted approach. When subjected to conditions of cold, dry air, plasmodia respond by slowing their locomotion (Saigusa et al. 2008). If this negative stimulus is applied in short bouts at regular intervals such as each hour, plasmodia would respond—after only three such intervals—by spontaneously slowing down locomotion on the fourth interval, even when the negative stimulus was not applied. Continued absence of the negative stimulus led to resumption of normal, sustained locomotion, but the same anticipatory response could be evoked, even six hours later, after a single application of cold dry air. These results indicate that Physarum possesses some cellular mechanism for memorising periodicity and recalling this periodicity at a later time. Rats have been shown to time intervals using a self-sustaining endogenous oscillator (Crystal 2006); hence it is possible (though yet untested) that Physarum utilises its own endogenous oscillations to time intervals.

### Learning

With the ability to store and retrieve information about past events, organisms have the potential to change their behaviour based on their recalled experience. If that behavioural change imparts a fitness benefit, then natural selection should favour those organisms. Many definitions would classify these organisms with the ability to learn (Ginsburg and Jablonka 2009). Hence, many recent studies have focussed on defining the learning capabilities of Physarum, beginning with one of the simplest forms of non-associative learning: habituation.

Habituation occurs when an organism decreases its response to a stimulus after repeated or prolonged exposure to that stimulus (Perry et al. 2013; Shettleworth 2009). Physarum plasmodia that are separated from access to a food source by a bridge of repellent quinine-agar show clear aversive responses to the quinine-agar (Boisseau et al. 2016). However, when this stimulus was repeated for 5 days, plasmodia gradually reduced their aversive response, habituating to the negative stimulus. This response was repeated with another repellent, caffeine, but quinine-habituated plasmodia did not reduce their aversive response to caffeine, and vice versa. By demonstrating response specificity, the researchers ruled out the potential confounding factors of fatigue or general sensory adaptation.

A follow-up study demonstrated that the learned response survived the process of cell–cell fusion with a clonemate plasmodium (Vogel and Dussutour 2016). By placing habituated and unhabituated plasmodia next to each other and allowing them to fuse, pseudopodia which crossed the repellent–agar bridge were just as likely to originate from the region of previously unhabituated biomass as the habituated region. While the authors describe this result as unhabituated plasmodia “directly acquir[ing] a learned behaviour from a habituated slime mould”, it is incorrect to label these regions as individual plasmodia after cell–cell fusion has occurred. Indeed, the authors state that “extensive protoplasmic mixing took place”, quickly rendering the fused clones as a single entity of mixed protoplasm from habituated and unhabituated donor plasmodia. A later study provided supporting evidence that the mechanism underlying habituation, and its transferability between plasmodia, is high levels of the repellent stimulus itself (in this case NaCl salt) being taken up into the cell and distributed throughout the protoplasm as a ‘circulating memory’ (Boussard et al. 2021). By contrast, Smith-Ferguson and colleagues (2022) found the opposite result in a study published a year later. Plasmodia repeatedly exposed to NaCl were more likely to avoid the salt. This appears to show an example of sensitisation, another form of learning. The researchers argue this could be due to a greater build-up of NaCl in the latter study; plasmodia in the earlier study had prolonged but non-repeated exposure to the stimulus.

While habituation has been characterised as the “simplest” (Hawkins and Kandel 1984; Rose and Rankin 2001) or “most ancient” (Van Duijn 2017) form of learning, the successful demonstration of any form of learning in a brainless organism is an achievement in itself. The question of learning in unicellular organisms was hotly debated in the early twentieth century, the result of which was the prevailing view that non-associative learning was possible for these ‘simple’ creatures, but not higher forms of associative learning such as Pavlovian conditioning. Later scientific attempts to disprove this notion were generally condemned on grounds of non-reproducibility or misinterpretation (see Gershman et al. (2021) for an excellent review of the topic). Recent success in demonstrating ‘entry-level’ learning abilities in Physarum begs the obvious question of whether Physarum is capable of associative learning. Such a demonstration could have widespread impacts (or maybe not, see “Discussion”).

Only one published study has claimed to demonstrate associative learning in Physarum (Shirakawa et al. 2011). As discussed in the subsequent literature (Dussutour 2021; Krause et al. 2022; Loy et al. 2021), and conceded by the authors themselves, the observed results have more parsimonious explanations. Pairing the food reward stimulus with the conditioned negative stimulus of low temperature, trained plasmodia were observed to move towards both stimuli, while untrained control plasmodia avoided the low-temperature option. Temperature affects many aspects of slime mould physiology, including metabolic rate, movement speed and rates of chemical uptake and sensing, and so could introduce confounding factors that could be interpreted as association. Loy et al. (2021) say the data presented “are not sufficient to assess the effectiveness of the conditioning treatment”. Dussutour (2021) posits that if learning did occur, the plasmodia are likely to have habituated to the low temperature. While the possibility of associative learning in non-neural organisms is daily becoming less extraordinary, common acceptance of the notion amongst animal cognition researchers requires extraordinary evidence, which has so far yet to be produced (but see Carrasco-Pujante et al. (2021) for recent evidence of associative conditioning in unicellular amoebae).

## Discussion

Physarum clearly possesses many of the hallmarks of cognition, including sensing, communication, navigation, decision-making, memory and learning. The increasing popularity of behavioural research in easy-to-use Physarum points to the slime mould emerging as a model for non-neural cognition. Doubtless, as further research progresses more abilities from the traditional cognitive tool-kit will be added to Physarum’s demonstrated repertoire.

Beyond adding demonstrations of further cognitive capabilities, future researchers also need to focus on understanding the intracellular mechanisms of cognitive behaviour in this protist. While there has been extensive measurement and modelling of the oscillatory system (Gao et al. 2019; Nakagaki et al. 1999; Wohlfarth-Bottermann 1979), the work to understand the molecular underpinnings of cognition has only just begun, most notably with the role of an intracellular signalling agent (Kramar and Alim 2021) and the role of chemical retention during habituation (Boussard et al. 2021). This is in stark contrast to the wealth of accumulated knowledge on the molecular machinery of neural cognitive systems. While seeking to close this knowledge gap, researchers must also pay attention to appropriate experimental design for testing cognition in organisms that often operate at such a different temporal and spatial scale to our own (for a detailed outline of these challenges, see Reid et al. 2015).

Classic models of cognitive processing have drawn a line between the cognitive and non-cognitive organisms based on the flow of sensorimotor information. Non-cognitive organisms are defined by reactions to external stimuli without internal feedback between the stimulus receptor and the site of action, while cognitive organisms are those that modulate the receptor via internal neural feedback from the site of action (Fuster and Bressler 2012; Reid and Latty 2016; von Uexküll 1926). The emerging trend of broader phylogenetic inclusivity in cognitive research has led to descriptions of other sensorimotor feedback systems that need not rely on neurons, such as the two-component signal transduction system of the bacterium *E. coli* (van Duijn 2006), and the coupled-oscillation system of Physarum (Reid and Latty 2016). Indeed, Baluška and Levin (2016) point out that neurons are ill-deserving of their reputation for “magical, unique” cognitive properties, because cognitive computations may arise from “the dynamics of networks of linked elements that propagate and integrate signals, and the ability to alter connectivity among those elements (network topology) based on prior activity.” This statement itself could serve as a description of Physarum and its mode of action.

Proponents of basal cognition remind us that neural networks evolved from far more ancient signalling pathways; neurons mainly optimised existing mechanisms for speed (Sterling and Laughlin 2015). Taking this perspective offers new avenues for understanding the evolution of not only cognition, but also of nervous systems themselves. Sensorimotor coordination, described as “the process by which organisms adaptively coordinate their sensors and effectors to optimize the external conditions for their metabolism and homeostasis” (van Duijn 2017), is an ancient strategy that enabled complex forms of cognition to evolve. It is also a popular hypothesis for why nervous systems evolved in the first place. This is in part attributed to size: tiny organisms can function adequately using sensorimotor systems based on cilia, while larger, more complex organisms require something like a nervous system to support their muscle-based locomotion. According to this ‘moving hypothesis’ (Llinás 2002), higher motility and larger size led to the exploitation of more heterogeneous environments and development of evolutionary arms races that fuelled a massive explosion in morphological and behavioural diversity. Physarum mirrors this pattern: it has high motility, including an extremely fast rate of cytoplasmic streaming (up to 1 mm/sec) coupled with an ability to reach enormous sizes for a single cell. Physarum’s large size is in part facilitated by its unique contractile mechanism of information transfer, which may in turn have enabled it to access more heterogeneous environments, leading to the exploitation of higher cognitive niches than your average protist.

Taking the more phylogenetically inclusive approach to cognition could benefit our understanding of extant decision-making systems as well. One of the most widely accepted models of decision making (the drift–diffusion model), is based on how neurons interact within the brain. ‘Evidence’ in favour of competing options (in the form of firing rate) builds in competing neurons, until one of them exceeds a decision threshold (Bogacz et al. 2006; Chittka et al. 2009; Livnat and Pippenger 2006). A strong analogy exists here with the oscillation system of Physarum (Reid and Latty 2016). More recently, oscillation patterns in Physarum plasmodia were observed to be highly dynamic, consisting of interlaced regular and irregular contraction patterns, similar to neural activity observed in nematodes and fruit flies (Fleig et al. 2022). These observations, coupled with the ‘non-adaptive’ examples of speed-accuracy trade-offs and irrational decision-making in Physarum (not to mention the evidence for cognitive capabilities in other non-neural taxa), provide strong evidence for fundamental principles of information processing and decision-making that span the majority, if not the entirety, of the phylogenetic tree (Reid and Latty 2016). Embracing this viewpoint could have significant and measurable impacts on the field of cognition. When cognitive science restricts its viewpoint to ‘brains and above’, it at best underestimates the diversity of strategies available, and at worst may remain blind to the real underlying mechanisms of cognition.

The elusive demonstration of associative learning in a non-neural organism is still a passionately sought goal of many labs around the world, with Physarum researchers only the most recent to join the race. The Physarum model system, due largely to its ease-of-use and robustness to experimentation, has indeed been consistently successful at puncturing previously held cognitive prejudices. This success certainly favours Physarum as a non-neural system that has the potential to demonstrate associative learning, but this is far from a foregone conclusion. Smith-Ferguson and colleagues (2022) conclude—probably correctly, given the bias against publication of negative results—that a number of attempts to show associative learning in a wide range of organisms have probably failed. If so, that raises a question: why? If information processing or even cognition are ubiquitous among taxa, why not associative learning? They argue that Physarum simply has no strong selective pressure necessitating any kind of learning mechanism more complex than habituation. However, they concede that Physarum’s inability to make associations has not been thoroughly tested.

If definitive proof of this ‘gold standard of the cognitive’ were to be found in Physarum, what would that mean for the field of cognition? It would certainly cement the ascribed importance of taking a more holistic approach to cognitive science across taxa, and would spur vigorous research interest into the mechanisms of learning in ‘non-neuralia’. However, it could just as likely be viewed as just another neat trick pulled by Physarum and contribute little to the trajectory of animal cognition research into the future. This is especially likely if cognition researchers relegate Physarum behavioural research to a separate, irrelevant domain through the clever use of definitions. Physarum behaviour has been classed as basal cognition (Lyon et al. 2021), embodied cognition (Cheng 2022), extended cognition (Sims and Kiverstein 2022), and minimal cognition (see Vallverdú et al. (2018) for a list of references demonstrating each of the ten biogenic principles of minimal cognition from Lyon (2006), across a wide swathe of Physarum research). While it is conventional and often necessary to strictly define domains of research, definitions can also be misused to exclude entire fields that do not sit well with the established narrative (equating to a ‘No true Scotsman’ fallacy). For this reason, Smith-Ferguson and Beekman (2020) use Physarum behavioural research to argue against using the term cognition at all, “in favour of discussing various forms of information processing”.

In the recent 25th Anniversary retrospective edition of Trends in Cognitive Sciences, the question was raised: “What would make cognitive science more useful?” (Lewis Jr 2022). Lewis identifies as a major hurdle the “large discrepancy between the homogeneous samples that our field studies and the diverse populations that exist in the broader world—discrepancies that distort our understanding of how minds work and why they work in the ways that they do.” This discrepancy has been known for decades (see the famous study by Henrich et al. (2010) on the disproportionate use of WEIRD samples in human psychology). While Lewis’ target was human populations and the human mind, his statement equally applies to all organisms and all minds. Recent research on Physarum and other ‘basal’ organisms has shown us that true understanding of how minds work and why requires greater understanding of the diversity of minds that exist in the broader natural world.

## Data Availability

Not applicable.
